# Floristic survey of vascular plants of the Parque Estadual da Pedra Selada, Rio de Janeiro, Brazil

**DOI:** 10.3897/BDJ.12.e129475

**Published:** 2024-10-04

**Authors:** Isabela Maciel Waga, Andrea Ferreira da Costa, Claudine Massi Mynssen, Eduardo Pinheiro Fernandez, Elsie Franklin Guimarães, Fernanda Saleme, George Azevedo de Queiroz, Guilherme Medeiros Antar, Haroldo Cavalcante de Lima, Hemily Oliveira Marques, Lara Serpa Jaegge Deccache, Leandro Jorge Telles Cardoso, Leandro Lacerda Giacomin, Maria Regina de V. de Vasconcellos Barbosa, Mario Gomes, Marli Pires Morim, Otávio Luis Marques da Silva, Pedro Fiaschi, Pedro Luís Rodrigues de Moraes, Rafaela Campostrini Forzza, Renon Santos Andrade, Thaís Andrade Ferreira Dória, Thiago Serrano de Almeida Penedo, Thuane Bochorny, Marcio Verdi

**Affiliations:** 1 Jardim Botânico do Rio de Janeiro, Rio de Janeiro, Brazil Jardim Botânico do Rio de Janeiro Rio de Janeiro Brazil; 2 Centro Nacional de Conservação da Flora, Rio de Janeiro, Brazil Centro Nacional de Conservação da Flora Rio de Janeiro Brazil; 3 IUCN SSC Brazil Plant Red List Authority, Rio de Janeiro, Brazil IUCN SSC Brazil Plant Red List Authority Rio de Janeiro Brazil; 4 Universidade Federal do Rio de Janeiro, Rio de Janeiro, Brazil Universidade Federal do Rio de Janeiro Rio de Janeiro Brazil; 5 Universidade Federal do Espírito Santo, São Mateus, Brazil Universidade Federal do Espírito Santo São Mateus Brazil; 6 Universidade Federal da Paraíba, João Pessoa, Brazil Universidade Federal da Paraíba João Pessoa Brazil; 7 Instituto de Pesquisas Ambientais do Estado de São Paulo, Sao Paulo, Brazil Instituto de Pesquisas Ambientais do Estado de São Paulo Sao Paulo Brazil; 8 Universidade Federal de Santa Catarina, Florianópolis, Brazil Universidade Federal de Santa Catarina Florianópolis Brazil; 9 Universidade Estadual Paulista Júlio de Mesquita Filho, Rio Claro, Brazil Universidade Estadual Paulista Júlio de Mesquita Filho Rio Claro Brazil; 10 Instituto Chico Mendes de Conservação da Biodiversidade, Bahia, Brazil Instituto Chico Mendes de Conservação da Biodiversidade Bahia Brazil; 11 Fundação Brasileira Desenvolvimento Sustentável, Rio de Janeiro, Brazil Fundação Brasileira Desenvolvimento Sustentável Rio de Janeiro Brazil

**Keywords:** Atlantic Forest, conservation, endemism, plant richness, threatened species

## Abstract

**Background:**

The Atlantic Forest is one of the most diverse and threatened phytogeographical domains in the world. Despite that, it includes regions with poor floristic knowledge, even in protected areas. Although the importance of protected areas in conserving the Atlantic Forest hotspot is undisputed, it is necessary to recognise the floristic richness of these areas to propose effective conservation actions. In this sense, online databases have proved to be a promising tool for compiling species lists with relevant biodiversity information. This study is based on the list of vascular plants of the "Parque Estadual da Pedra Selada", published in the "Catálogo de Plantas das Unidades de Conservação do Brasil". It summarises the species richness, endemism and conservation status of this protected area.

**New information:**

The published list of vascular plants was based on data obtained from herbarium collections available in online databases. A total of 303 species have been recorded for the "Parque Estadual da Pedra Selada," of which 297 are native to Brazil, 78 are endemic to the Brazilian Atlantic Forest, and seven are endemic to the State of Rio de Janeiro. More than 60% of the species are woody, and more than 40% are trees. Eight threatened species (Endangered – EN and Vulnerable – VU), of which five are endemic to the State of Rio de Janeiro, are housed in this protected area. One species was classified as Data Deficient (DD). Our results increase the knowledge of the Atlantic Forest flora in the State of Rio de Janeiro and support effective conservation planning for this protected area.

## Introduction

Countries that house tropical forests have some of the most biodiverse and endangered ecosystems in the world (Alroy 2017, Zwiener et al. 2017). In Brazil, the Atlantic Forest phytogeographical domain – hereinafter referred to as Atlantic Forest – stands out as one of the most biodiverse and the one with the highest rates of endemism (Werneck et al. 2011, BFG 2018, BFG 2021, Marques and Grelle 2021). The Atlantic Forest, however, has undergone an intense process of habitat loss and fragmentation, resulting in a drastic reduction in native vegetation cover (Ribeiro et al. 2009). Leading to the current scenario where only 12.4% to 28% of its original area remains (Ribeiro et al. 2009, Rezende et al. 2018, SOS Mata Atlântica, INPE 2020), it is distributed in small and disconnected remaining forest fragments. The mapped native vegetation is likely composed mainly of edge-affected or secondary vegetation, disconnected from large fragments, with a substantial portion located outside designated protected areas (Rezende et al. 2018).

Amongst the 17 Brazilian states in the Atlantic Forest domain, Rio de Janeiro (RJ) has a great diversity of landscapes (Costa et al. 2009). The State is fully inserted in the Atlantic Forest and is characterised by six phytophysiognomies shaped by relief and climate variations (Dantas 2000):

(i) Dense Ombrophilous Forest (Lowland, Sub-Montane, Montane, and High-Montane),

(ii) Semi-deciduous Seasonal Forest (Lowland, Sub-Montane, Montane),

(iii) Vegetation with Marine Influence (sandbanks),

(iv) Vegetation with Fluviomarine Influence (mangroves),

(v) Ecological Refuges (high-altitude grasslands and inselbergs), and

(vi) Wooded Steppe-Savannah (IBGE 2012).

Rio de Janeiro, together with Bahia, Minas Gerais and São Paulo, leads the list of states with the greatest floristic diversity of several taxonomic groups (BFG 2021). Rio de Janeiro houses 1,106 endemic land plant species (Embryophyte) (Flora e Funga do Brasil 2023) and is also a protagonist in the number of threatened species with high conservation value (Martinelli et al. 2018, Martins et al. 2018). The pattern of occupation and land use in the State is marked by five economic cycles, based on the exploitation of natural resources (Dean 1996, Costa 2022), the same as that observed for the Atlantic Forest as a whole. Due to the historical process of habitat loss and fragmentation, the natural vegetation cover in the State is already vulnerable, but several human activities continue to threaten its biodiversity conservation (Pougy et al. 2018).

In this scenario, there is a need to invest in field expeditions to advance the floristic knowledge of the protected areas (Coelho et al. 2017, Oliveira et al. 2017). The progress made in recent years with online national biodiversity databases, such as Reflora (2023), Jabot (JBRJ - Instituto de Pesquisas Jardim Botânico do Rio de Janeiro 2022), ProFlora (Calfo et al. 2021) and INCT Herbário Virtual da Flora e dos Fungos do Brasil (speciesLink 2022) is undeniable. These databases, however, were developed with specific proposals, making it difficult to build up data on the flora of protected areas (Moreira et al. 2020). In addition, several groups of plants are still poorly known, affecting the floristic knowledge of the areas where they occur. To fill these gaps, the "Catálogo de Plantas das Unidades de Conservação do Brasil" was launched online in 2018 to contribute to the access and dissemination of information on the floristic knowledge of the Brazilian protected areas (Moreira et al. 2020). It allows users to access images of specimens of most species and to check the conservation status of every listed species, following the Brazilian National Center for Plant Conservation (CNCFlora/JBRJ, acronym in Portuguese for "Centro Nacional de Conservação da Flora" of "Jardim Botânico do Rio de Janeiro"). In this study, we provide and discuss the species richness, endemism and conservation status of the species in the "Parque Estadual da Pedra Selada" (PEPS).

## Sampling methods

### Study extent

**Figure 1.**
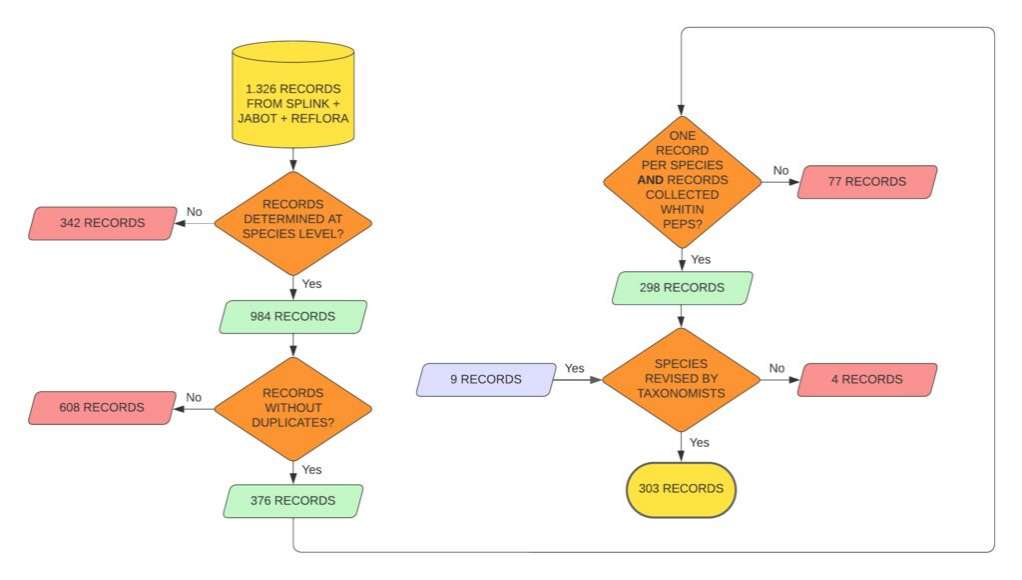
Workflow for data cleaning and preparation of the species list for "Parque Estadual da Pedra Selada", Brazil. The specimens kept on the list are shown in green, while the specimens removed are shown in red. The specimens that were later included by a taxonomist are shown in blue.

The list of vascular plants collected in the PEPS was generated from the compilation of data obtained from four databases: JABOT GERAL, JABOT RB, Herbário Virtual REFLORA and INCT Herbário Virtual da Flora e dos Fungos, hereafter speciesLink. The data were compiled in September 2022, with the filter location = "Pedra Selada". Searches returned a total of 1,326 specimens (JABOT GERAL = 214; JABOT RB = 490; REFLORA = 490; speciesLink = 132; Fig. 1). To obtain a list of species, we selected only specimens identified at species level: JABOT GERAL = 154 (undetermined = 60), JABOT RB = 348 (undetermined = 142), REFLORA = 368 (undetermined = 122) and speciesLink = 114 (undetermined = 18; Fig. 1).

We then removed duplicates based on the collector’s name, number and year of collection and selected one record per species, prioritising those records with digitised specimens. We also excluded records whose locations did not fall within the area of the PEPS (Fig. 1). Finally, we used the online tool Plantminer species (Carvalho et al. 2010) to update species names according to Flora e Funga do Brasil (2023). After these corrections, a preliminary list of 298 species was checked and validated by taxonomists using images available in the online databases. Intraspecific taxonomic categories and hybrids were not considered. When the taxonomist changed the identification of a species, at least one specimen of this species was updated in the JABOT and REFLORA databases. The final checklist of the vascular plants of the PEPS was published by Waga et al. (2022) and is available in the "Catálogo de Plantas das Unidades de Conservação do Brasil".

**Origin, endemism and conservation status**: Information about the origin (native, introduced or uncertain) and endemism of the species to the Brazilian Atlantic Forest follows Flora e Funga do Brasil (2023). The conservation status of the species is in accordance with the CNCFlora/JBRJ database, which serves as the IUCN SSC Brazil Plant Red List Authority (IUCN SSC BP-RLA) and provides the Official National Red List published by MMA Ordinance No. 148/2022.

**Life forms**: We adopted six categories, summarised from Flora e Funga do Brasil (2023): trees, shrubs, lianas, herbs, palms and bamboos. When there was more than one life form, we chose the most frequent in the herbarium records for PEPS, as proposed by Moreira et al. (2020). This information was also retrieved through Plantminer (Carvalho et al. 2010).

## Geographic coverage

### Description

**Figure 2.**
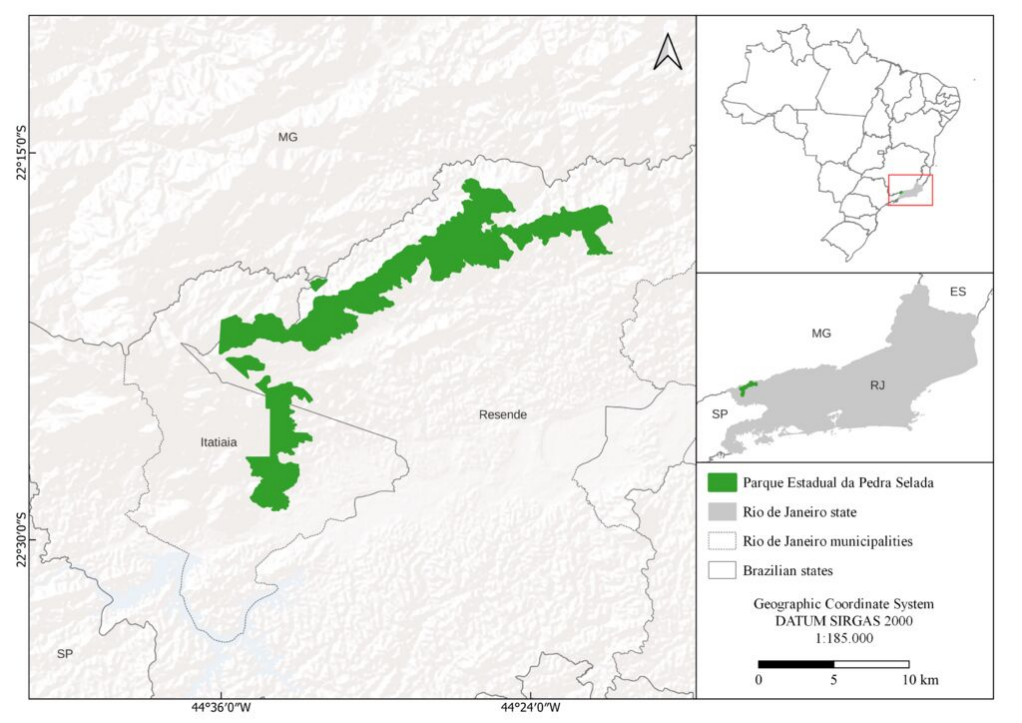
Location map of the "Parque Estadual da Pedra Selada", Rio de Janeiro, Brazil.

The PEPS is part of the Mantiqueira Mosaic of Protected Areas. Created on 15 June 2012, by State Decree No. 43,640, it presents an area of 8,036 hectares extending across the municipalities of Resende and Itatiaia, in the southwest region of the State of Rio de Janeiro (Fig. 2). The dominant forest physiognomy is the Montane Dense Ombrophilous Forest, which occurs between 500 and 1,500 m above sea level (a.s.l.) and covers around 83.5% of the PEPS area. High-altitude grasslands are found at elevations above 1,800 m and are characterised by herbaceous rupicolous vegetation, as in the case of "Pico da Pedra Selada" (Fig. 3).

The PEPS presents four climatic types, according to the Köppen-Geiger classification: Cwb (subtropical highland with dry winter and mild summer, generally above 1,600 m a.s.l.), Cwa (subtropical with dry winter and hot summer), Cfb (humid temperate with mild summer) and Cfa (humid subtropical with hot summer) (Alvares et al. 2013). The average annual temperature varies between −5°C and 30°C according to the elevation, which ranges from 420 m to 2,100 m a.s.l. The PEPS presents mountainous and rugged relief with the presence of Litholic Neosols and Humic Cambisols soils (Detzel et al. 2017).

### Coordinates

−22.485 and −22.260 Latitude; −44.325 and −44.602 Longitude.

## Taxonomic coverage

### Description

The plant list of the PEPS includes 303 species, 210 genera and 81 families, of which 282 species, 190 genera and 70 families are angiosperms, 20 species, 19 genera and 10 families are ferns and lycophytes and one species is a gymnosperm (*Podocarpuslambertii* Klotzsch ex Endl.; Fig. 4a). The richest families in PEPS are: Fabaceae (31 spp., 10.23%), Asteraceae (25 spp., 8.25%), Melastomataceae (18 spp., 5.94%), Rubiaceae (17 spp., 5.61%), Solanaceae (12 spp., 3.96%), Bromeliaceae and Myrtaceae (10 spp., 3.30% each), Piperaceae, Lauraceae, and Euphorbiaceae (3%, 9 spp. each; Fig. 4b). These families represent 40.59% (123 spp.) of the species found in the area. Four of them have been reported as the five richest angiosperm families in Rio de Janeiro State (Coelho et al. 2017) and six are included in the top ten richest angiosperm families in Brazil and in the Atlantic Forest (BFG 2021). On the other hand, 33 families are represented by a single species. The richest genera are: *Solanum* L. and *Piper* L. (8 spp., 2.64% each), *Miconia* Ruiz & Pav. (7 spp., 2.31%), *Mimosa* L. (6 spp., 1.98%), *Ocotea* Aubl. and *Begonia* L. (5 spp., 1.65% each), *Myrcia* DC., *Leandra* Raddi, *Croton* L. and *Baccharis* L. (4 spp., 1.32% each; Fig. 4c), representing 18.15% of the species.

In relation to ferns and lycophytes, the richest families are Polypodiaceae (7 spp.), Pteridaceae (3 spp.), Aspleniaceae and Lycopodiaceae (2 spp.; Fig. 4d). All these fern families, except Lycopodiaceae, were pointed out as the richest in Rio de Janeiro and in Brazil (Coelho et al. 2017, Prado et al. 2015). The richest genus is *Asplenium* L. with two species, all the others having only one species.

## Traits coverage

The PEPS presents a gradient of vegetation types that will vary from Montane Dense Ombrophilous Forest to High-Montane Dense Ombrophilous Forest and there are also records of Altitude Grasslands (Waga et al. 2022). In this diversity of environments, naturalised species have been recorded, as well as native species, species endemic to Rio de Janeiro and species classified as threatened.

### Origin, endemism and conservation status

The PEPS vascular plant list includes 297 native species and six naturalised species, of which five belong to Asteraceae. All species of gymnosperms, ferns and lycophytes are native. We found 78 (25.74%) species endemic to the Atlantic Forest, 74 angiosperms and four ferns and lycophytes. The family with more endemic species for the Atlantic Forest is Melastomataceae (11 spp., 3.63%), followed by Solanaceae (9 spp., 2.97%), Euphorbiaceae and Rubiaceae (5 spp., 1.65% each) and Bromeliaceae and Fabaceae (4 spp., 1.32% each). Seven of the endemic species of angiosperm registered in the PEPS are also endemic to Rio de Janeiro (Table 1).

The PEPS is home to 60 species that had their conservation status assessed following the process of CNCFlora/JBRJ. We registered eight threatened species, of which five were considered "Endangered" (EN) and three "Vulnerable" (VU). One more species was categorised as "Data Deficient" (DD; Fig. 5). Amongst the threatened species, five are endemic to the State of Rio de Janeiro (Table 1). One other species endemic to Rio de Janeiro was considered "Near Threatened" (NT). Additionally, the PEPS hosts six other species that were assessed as NT and 51 as "Least Concern" (LC).

### Life forms

About 43% (122 spp.) of angiosperms are trees, 25% shrubs (71 spp.), 20% herbs (57 spp.), 10% lianas (29 spp.), 0.7% palms (2 spp.) and 0.3% bamboos (1 sp.). The only gymnosperm record (*Podocarpuslambertii*) is a tree and all ferns and lycophytes are herbs.

## Temporal coverage

### Notes

The specimens collected by L.J.T. Cardoso, during the Project "Diagnóstico para o Plano de Manejo do Parque Estadual da Pedra Selada", are from 2015 and worth mentioning, as well as the collections of C. Baez and M. Verdi, for the Project "Inventário Florístico em UCs Estaduais – CNCFlora/JBRJ and SEAS-RJ" from 2018. All of them are deposited in the Herbarium RB.

## Usage licence

### Usage licence

Creative Commons Public Domain Waiver (CC-Zero)

## Data resources

### Data package title

Floristic survey of vascular plants of the Parque Estadual da Pedra Selada, Rio de Janeiro, Brazil

### Resource link

https://doi.org/10.5281/zenodo.11281100

### Alternative identifiers

https://doi.org/10.15468/f8434e; https://ipt.pensoft.net/resource?r=a-dataset-of-vascular-plant-species-in-parque-estadual-da-pedra-selada-rio-de-janeiro-brazil

### Number of data sets

1

### Data set 1.

#### Data set name

Checklist of vascular plant species in Parque Estadual da Pedra Selada

#### Data format

tsv

#### Download URL

https://zenodo.org/records/11281100/files/dataset_pedra_selada_state_park_v5.tsv?download=1

#### Description

Dataset published by Waga et al. (2024) contains information about the species of vascular plants in "Parque Estadual da Pedra Selada". It contains 303 species of vascular plants occurring in the Dense Ombrophilous Forest and highlights endemic species of the Atlantic Forest and the IUCN risk of extinction categories according to CNCFlora/JBRJ. These data are also available on GBIF - Verdi (2024).

**Data set 1.** 

Column label	Column description
occurrenceID	A unique identifier code for each record.
collectionCode	Database where the specimen can be found.
institutionCode	Hebarium of origin of the cited specimen.
basisOfRecord	The specific nature of the data record.
catalogNumber	Specimen reference code in the herbarium.
phylum	The full scientific name of the division in which the taxon is classified.
family	The full scientific name of the family in which the taxon is classified.
scientificName	Full name of the taxon in accordance with the Flora e Funga do Brazil.
recordedBy	Main collector of the specimen.
recordNumber	Main collector number of the specimen.
eventDate	The date when the event was recorded.
country	Country where the the specimen was recorded.
countryCode	Code of the country where the specimen was recorded.
stateProvince	The name of the next smaller administrative region than country (state, province, canton, department, region etc.).
municipality	The full name of the next smaller administrative region than county (city, municipality etc.).
verbatimLocality	The original textual description of the place.
decimalLatitude	Latitude of the point of the specimen recorded.
decimalLongitude	Longitude of the point of the specimen recorded.
geodeticDatum	The ellipsoid, geodetic datum or spatial reference system (SRS) upon which the geographic coordinates given in decimal Latitude and decimal Longitude are based.
coordinateUncertaintyInMetres	The horizontal distance (in metres) from the given decimal Latitude and decimal Longitude describing the smallest circle containing the whole of the Location.
establishmentMeans	Statement about whether a taxon has been introduced to a given place and time through the direct or indirect activity of modern humans.
endemism	Endemism of the species for the Mata Atlantica domain, based on the data of endemism and phytogeographic domain of the species obtained in Flora e Funga do Brazil.
conservationStatus	IUCN Red List category based on CNCFlora/JBRJ assessment.

## Additional information

The list of vascular plants of the "Parque Estadual da Pedra Selada" increases the knowledge of the Atlantic Forest flora of the State of Rio de Janeiro and supports an effective conservation plan for this protected area. We registered in the area 78 species endemic to the Atlantic Forest phytogeographical domain and seven endemics to Rio de Janeiro, of which five are threatened. These species should have specific actions for recovery included in the management plan of the PEPS. We highlight the need to increase collection efforts in the PEPS as well as in other protected areas in Rio de Janeiro.

## Figures and Tables

**Figure 3a.**
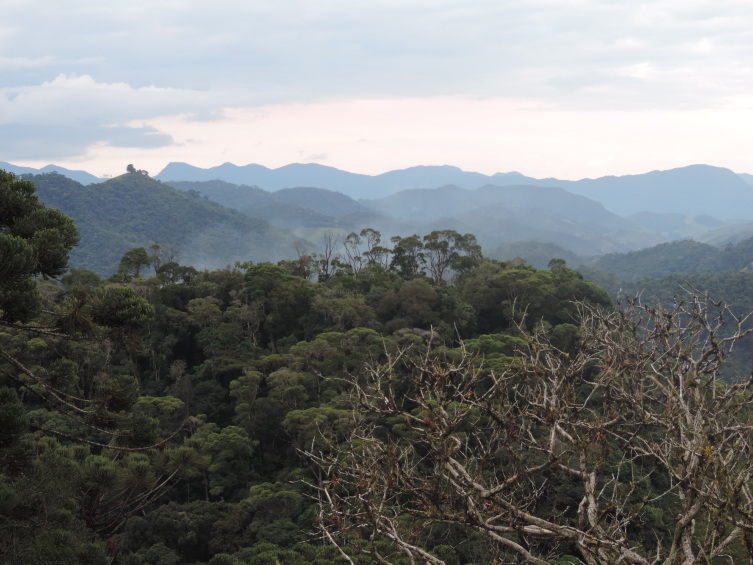
Montane Dense Ombrophilous Forest in PEPS;

**Figure 3b.**
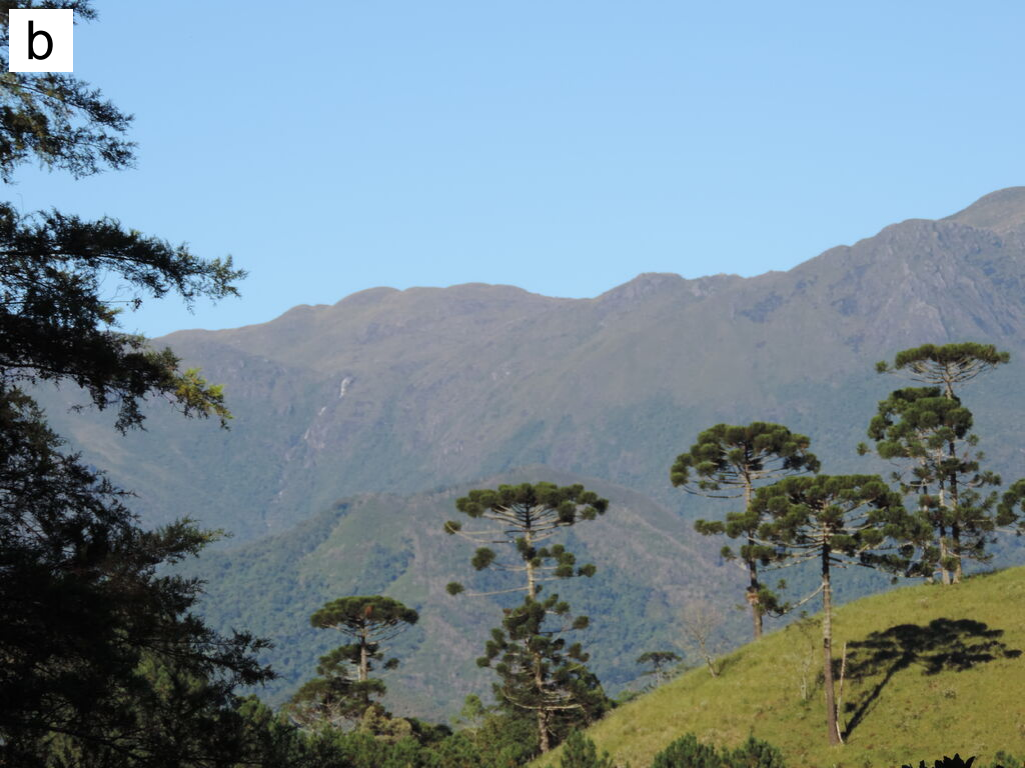
High-altitude grasslands in PEPS and *Araucariaangustifolia* (Bertol.) Kuntze trees in the vicinity;

**Figure 3c.**
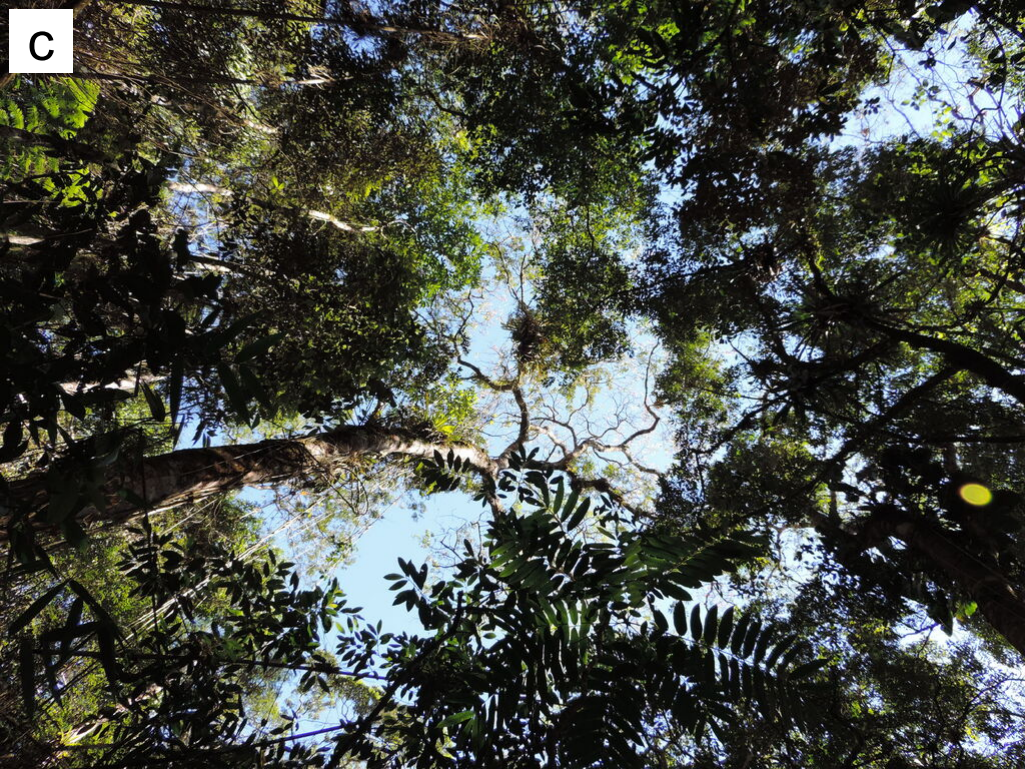
Canopy viewed from within the forest at PEPS;

**Figure 3d.**
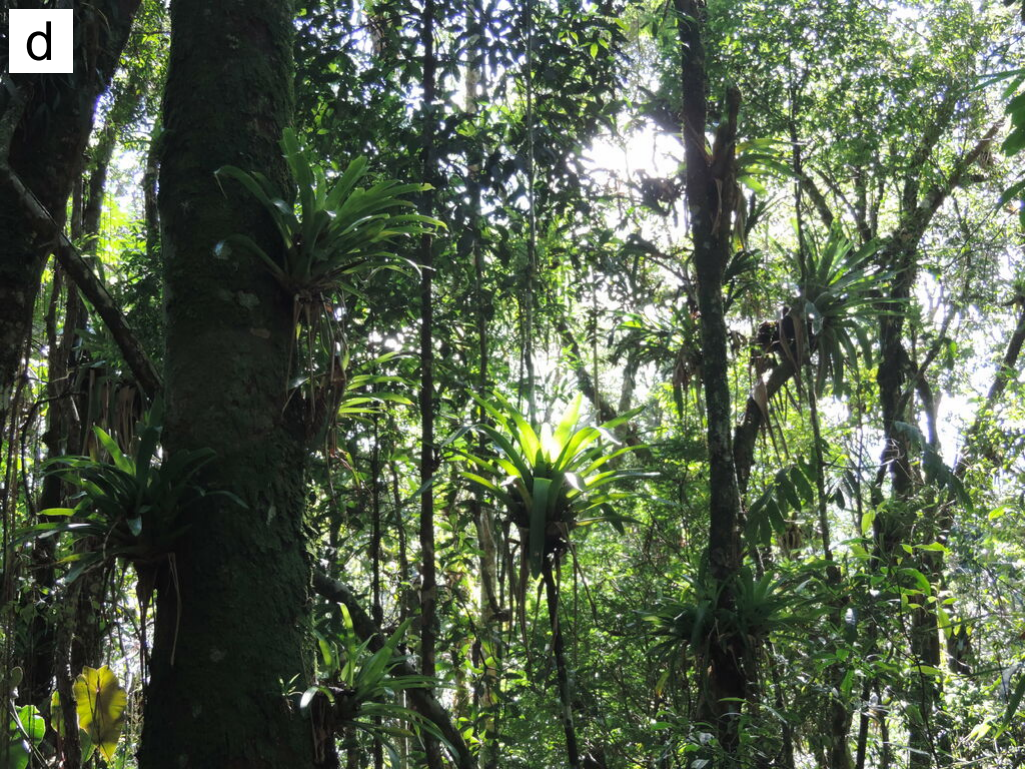
Abundance of epiphytic Bromeliaceae within the forest at PEPS.

**Figure 4a.**
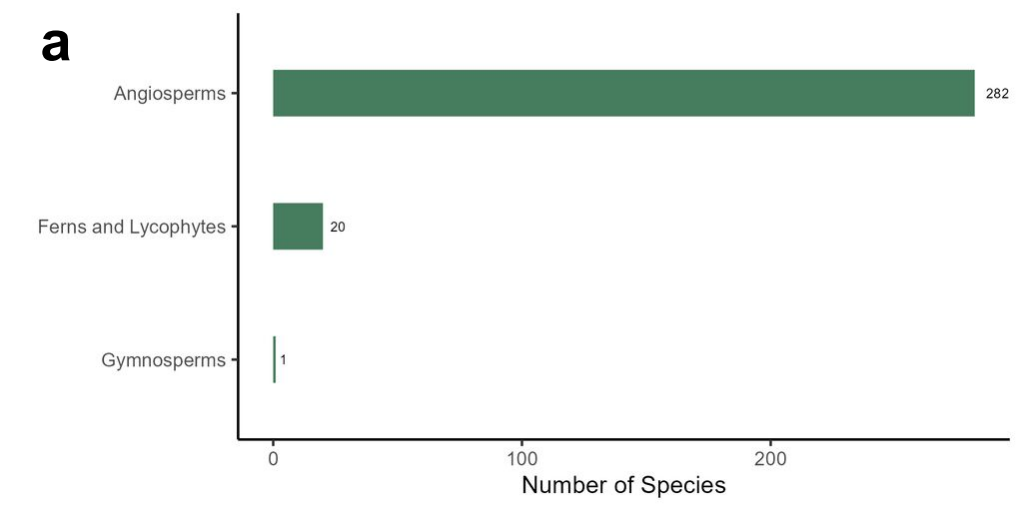
Richness of the plant groups from PEPS;

**Figure 4b.**
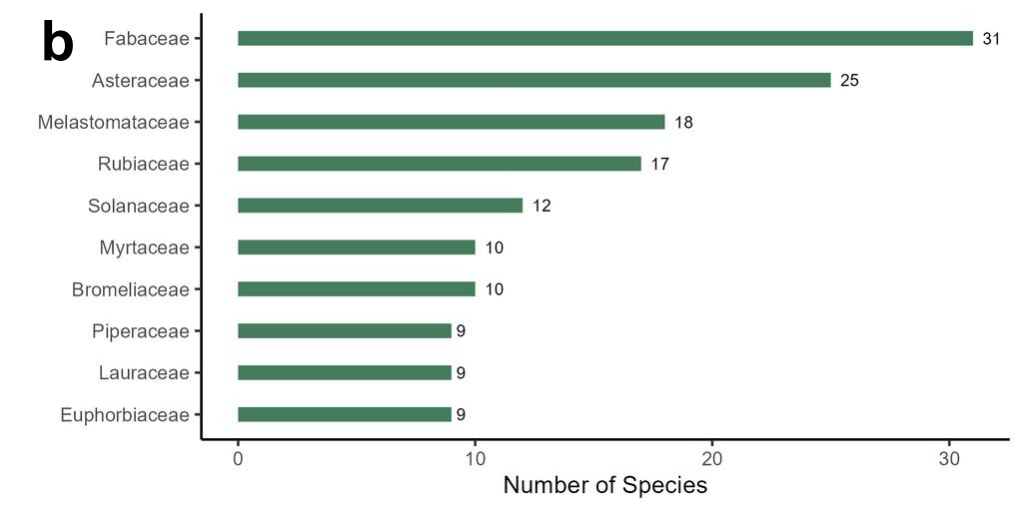
Richest families of angiosperms from PEPS;

**Figure 4c.**
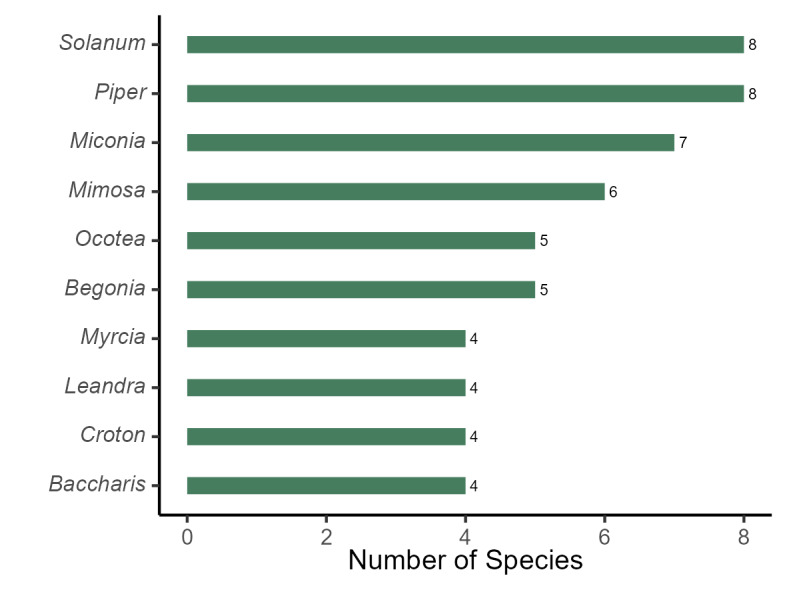
Richest genera of angiosperms from PEPS;

**Figure 4d.**
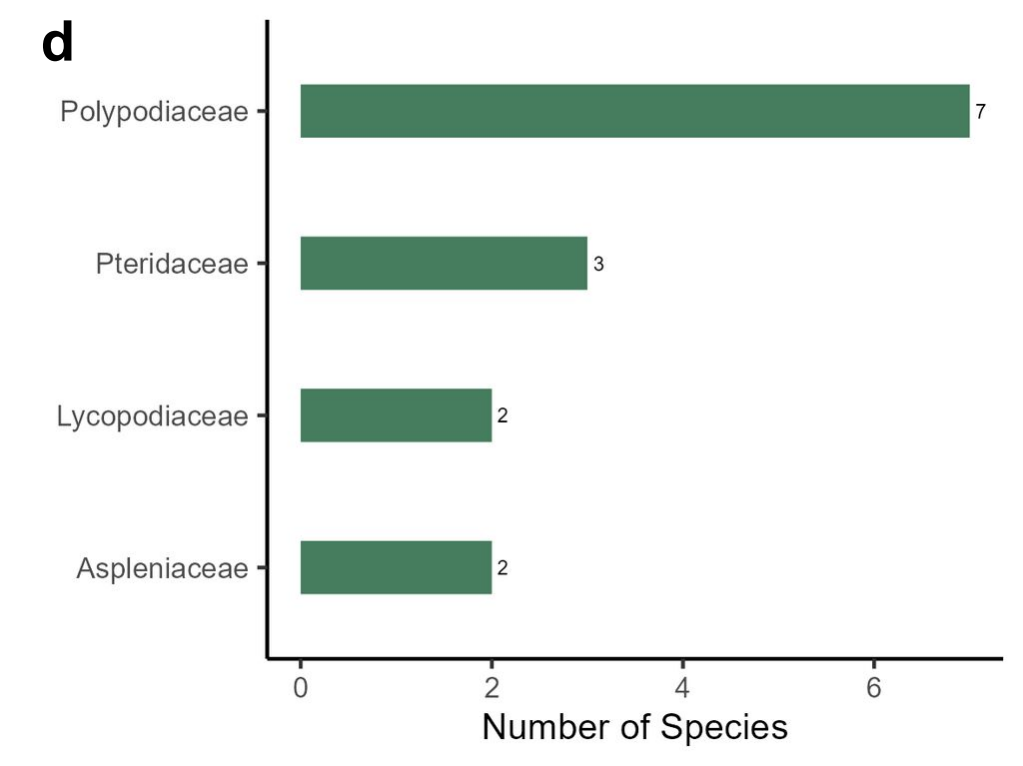
Richest families of ferns and lycophytes from PEPS.

**Figure 5a.**
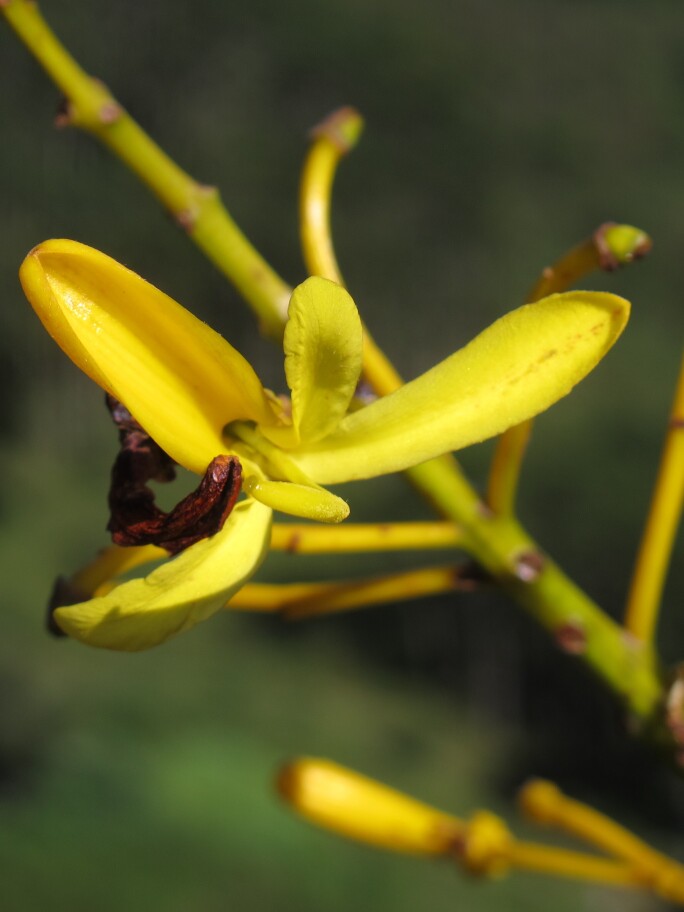
*Vochysiaglazioviana* Warm. (Vochysiaceae) – Endangered (EN). Photo: Leandro Cardoso;

**Figure 5b.**
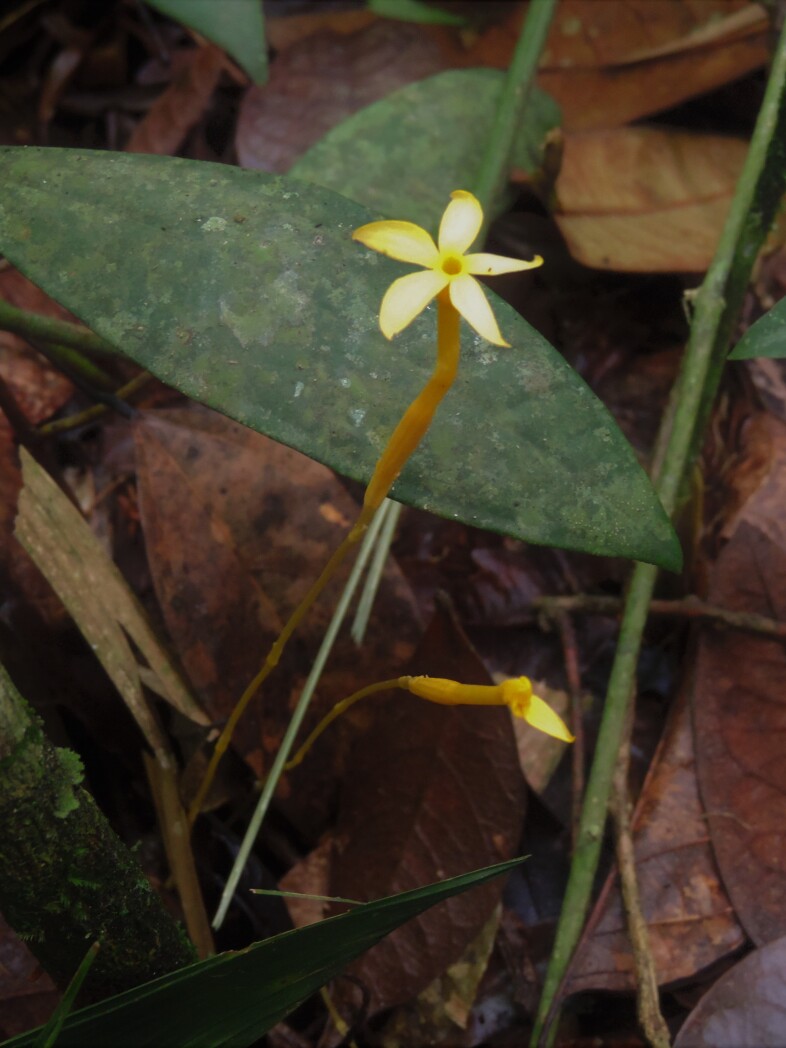
*Voyriaaphylla* (Jacq.) Pers. (Gentianaceae) – Data Deficient (DD). Photo: Eduardo Fernandez;

**Figure 5c.**
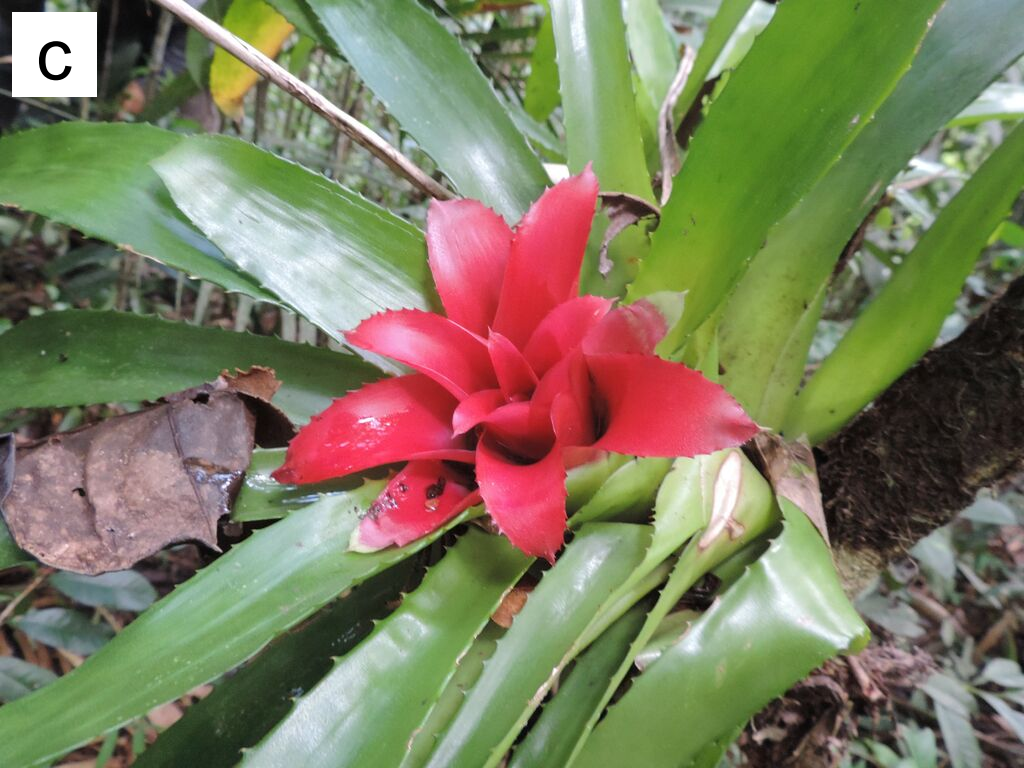
*Canistropsismarceloi* (E.Pereira & Moutinho) Leme (Bromeliaceae) – Vunerable (VU). Photo: Eduardo Fernandez.

**Table 1.** List of threatened and Data Deficient species registered in "Parque Estadual da Pedra Selada," Brazil and categories of other species endemic to Rio de Janeiro registered in the area, according to CNCFLora/JBRJ database (EN = Endangered, VU = Vulnerable and DD = Data Deficient).

**Family**	**Species**	**Category**	**Endemic to Rio de Janeiro**
Araceae	*Anthuriumparvum* N.E.Br.	NT	endemic
Araliaceae	*Dendropanaxnebulosus* Fiaschi & Jung-Mend.	EN	not endemic
Bromeliaceae	*Canistropsismarceloi* (E.Pereira & Moutinho) Leme	VU	endemic
Clusiaceae	*Tovomitopsissaldanhae* Engl.	-	endemic
Fabaceae	*Poecilanthefluminensis* Meireles & H.C.Lima	VU	endemic
Gentianaceae	*Voyriaaphylla* (Jacq.) Pers.	DD	not endemic
Meliaceae	*Cedrelafissilis* Vell.	VU	not endemic
Myrtaceae	*Campomanesiahirsuta* Gardner	EN	endemic
Onagraceae	*Fuchsiaglazioviana* Taub.	EN	endemic
Rubiaceae	*Psychotriaulei* Standl.	EN	endemic
Vochysiaceae	*Vochysiaglazioviana* Warm.	EN	not endemic
